# High Energy Density Anthraquinone-Based Redox Flow Batteries: From Molecular Engineering to Advanced Redox-Targeting Reaction

**DOI:** 10.3390/ma19204389

**Published:** 2026-10-19

**Authors:** Ge Yin, Yinuo Li, Zhiqian Wan, Xueli Sun, Sida Rong, Sheng Wen, Zhenming Zhang, Yuanzhi Gao, Ya Ji

**Affiliations:** 1 State Key Laboratory of Low-Carbon Smart Coal-Fired Power Generation and Ultra-Clean Emission, China Energy Science and Technology Research Institute Co., Ltd., Nanjing 210023, China20125642@ceic.com (S.W.);; 2 China-UK Low Carbon College, Shanghai Jiao Tong University, Shanghai 201306, Chinawanzhiqian@sjtu.edu.cn (Z.W.);

**Keywords:** redox flow batteries, anthraquinones, molecular engineering, redox-targeting reaction, high energy density

## Abstract

Anthraquinone-based redox flow batteries (AQ-RFBs) provide a tunable platform for molecularly engineered long-duration energy storage with high cell voltage and fast redox kinetics. However, high energy density and long-term stability of AQ-RFBs remain challenging. This review presents a systematic path from molecular engineering to redox-targeting flow batteries (RTFBs) for high-energy density anthraquinone-based redox flow batteries. Functional groups that regulate frontier molecular orbitals, redox potentials, and reaction kinetics are discussed for enhancing aqueous solubility and stability. Degradation pathways of anthraquinones are evaluated with spectroscopic, electrochemical, and computational methods in this work. Beyond molecular engineering strategies for anthraquinone-based redox flow batteries, novel redox-targeting (RT) flow battery systems are comprehensively reviewed. Anthraquinone-based RT flow batteries break the boundary between liquid and solid energy storage modes, greatly enhancing the energy density of AQ-based flow batteries and reducing AQ degradation at low concentrations. Redox-targeting concepts, AQ-based RTFB configurations, and advanced characterizations are well discussed. Finally, the challenges and outlooks of AQ-based flow batteries are stated for future research. In summary, this review highlights the development of anthraquinone-based flow batteries in terms of molecular engineering and redox-targeting design, providing valuable insights into high-energy-density aqueous organic redox flow batteries for wide applications.

## 1. Introduction

Accelerating the global transition toward decarbonized energy infrastructures demands large-scale electrical energy storage systems capable of mitigating the intermittency of renewable wind and solar power generation [1,2,3]. Grid-scale stabilization requires long-duration energy storage technologies that provide extended multi-hour discharge capabilities, modular scalability, and operational safety [4,5]. Among available electrochemical devices [6,7], redox flow batteries (RFBs) occupy a privileged position because their structural architecture physically segregates the power-generating stack from the energy-storing liquid reservoirs [8,9,10]. This decoupling permits independent scaling of power ratings and total energy capacity [11,12], presenting an optimal operational profile for stationary utility-scale applications [13,14]. All-vanadium RFBs (VRFBs) remain the most commercially exploited chemistry [15], but their deployment is restricted by vanadium cost and supply concentration [16], volatile raw-material pricing, membrane fouling [17], and corrosive sulfuric acid media [18].

Aqueous organic RFBs, particularly anthraquinone-based systems, offer an attractive route to overcome these constraints by using earth-abundant elements such as carbon, hydrogen, oxygen, and nitrogen [19,20]. Molecular engineering of organic electroactive scaffolds enables fine adjustment of redox potentials, reaction kinetics, and environmental compatibility across a broad pH spectrum [21,22]. Among various organic candidates, anthraquinone (AQ) derivatives serve as prominent negative electrolyte materials, largely owing to their favorable two-electron redox kinetics, low formal potentials, and high natural abundance [23,24]. Foundational research established by Aziz and coworkers demonstrates that functionalizing anthraquinone cores with sulfonic acid or hydroxyl groups allows reversible operation in acidic and alkaline media with exceptional electrochemical reversibility [25]. Subsequent high-throughput theoretical screening confirms that the formal potentials of quinones can be systematically adjusted through rational substituent positioning on the aromatic ring, establishing anthraquinones as versatile scaffolds for safe aqueous electrical energy storage [26]. Despite these attractive electrochemical attributes, aqueous organic flow batteries based on anthraquinone anolytes face a fundamental technological bottleneck in their limited volumetric energy density, which remains substantially lower than that required for space-constrained installations [27]. The root cause of this limitation lies in the moderate water solubility of polycyclic aromatic quinones, whose rigid planar backbones exhibit strong crystal lattice packing and intense intermolecular π-π stacking interactions. When synthetic chemists introduce dense ionic groups or flexible polyethylene glycol side chains to force solubility toward highly concentrated regimes, new physical and chemical penalties arise [28]. Highly concentrated electrolytes suffer from exponential viscosity increases and depressed ionic conductivities, which increase pumping energy loss and cell transport polarization [29]. Crowding reduced anthrahydroquinones promotes bimolecular degradation pathways during deep cycling [30]. Reduced quinones undergo proton-coupled tautomerization into active anthrone intermediates that spontaneously dimerize into electrochemically inactive bisanthrones, creating permanent capacity decay that compromises cell lifetime [31]. Consequently, homogeneous aqueous electrolytes reach an intrinsic thermodynamic limit where attempts to enhance volumetric energy density trigger accelerated chemical failure.

To break past this physical solubility limit without incurring concentrated molecular degradation, the energy storage community explores a fundamental paradigm shift through redox-targeting flow batteries [32]. First conceptualized by Wang and coworkers, the redox-targeting concept fundamentally redefines the role of dissolved electroactive molecules by transitioning fluidic systems into heterogeneous solid–liquid hybrid energy storage mode [33]. Instead of acting as the sole charge reservoir, a dilute dissolved anthraquinone circulates through the electrochemical stack to function as a fast catalytic electron mediator, shuttling electrons between porous carbon electrodes and stationary, high-capacity solid materials housed in external reservoirs [34]. By decoupling electrical energy storage from liquid solubility limits, the overall volumetric capacity becomes governed by the packing density of the solid booster rather than fluid saturation concentration [35]. Recent breakthroughs demonstrate that coupling anthraquinone mediators with tailored organic polymers delivers unprecedented performance gains. In neutral aqueous solution, single-molecule redox-targeting systems utilizing 9,10-anthraquinone-2,7-disulphonic acid (2,7-AQDS) successfully drive insoluble polyimide to achieve substantial capacity enhancements over homogeneous counterparts through hydrogen-bond-mediated charge transfer [36]. Furthermore, designing single-molecule systems with dual-potential plateaus enables 2,7-AQDS to drive poly(2,5-dihydroxy-1,4-benzoquinone-3,6-methylene) with remarkable solid-state material utilization and prominent volumetric energy density expansion [37]. In alkaline systems, pairing durable 1,5-dihydroxyanthraquinone with poly(anthraquinonyl sulfide) granules buffers the solution state of charge, suppressing dimerization and achieving prolonged temporal stability with negligible capacity fading [38]. Meanwhile, high-capacity heterocyclic scaffolds like tribenzophenazine-10,15-dione demonstrate robust chemical endurance in alkaline media [39], and predictive quantum-mechanical calculations accurately guide the energetic alignment of 1,8-dihydroxyanthraquinone-2,7-disulfonic acid (1,8-DHAQDS) with poly(N-anthraquinoyl pyrrole) through explicit hydrogen-bonding models to achieve notable capacity multiplication [40].

**Figure 1:**
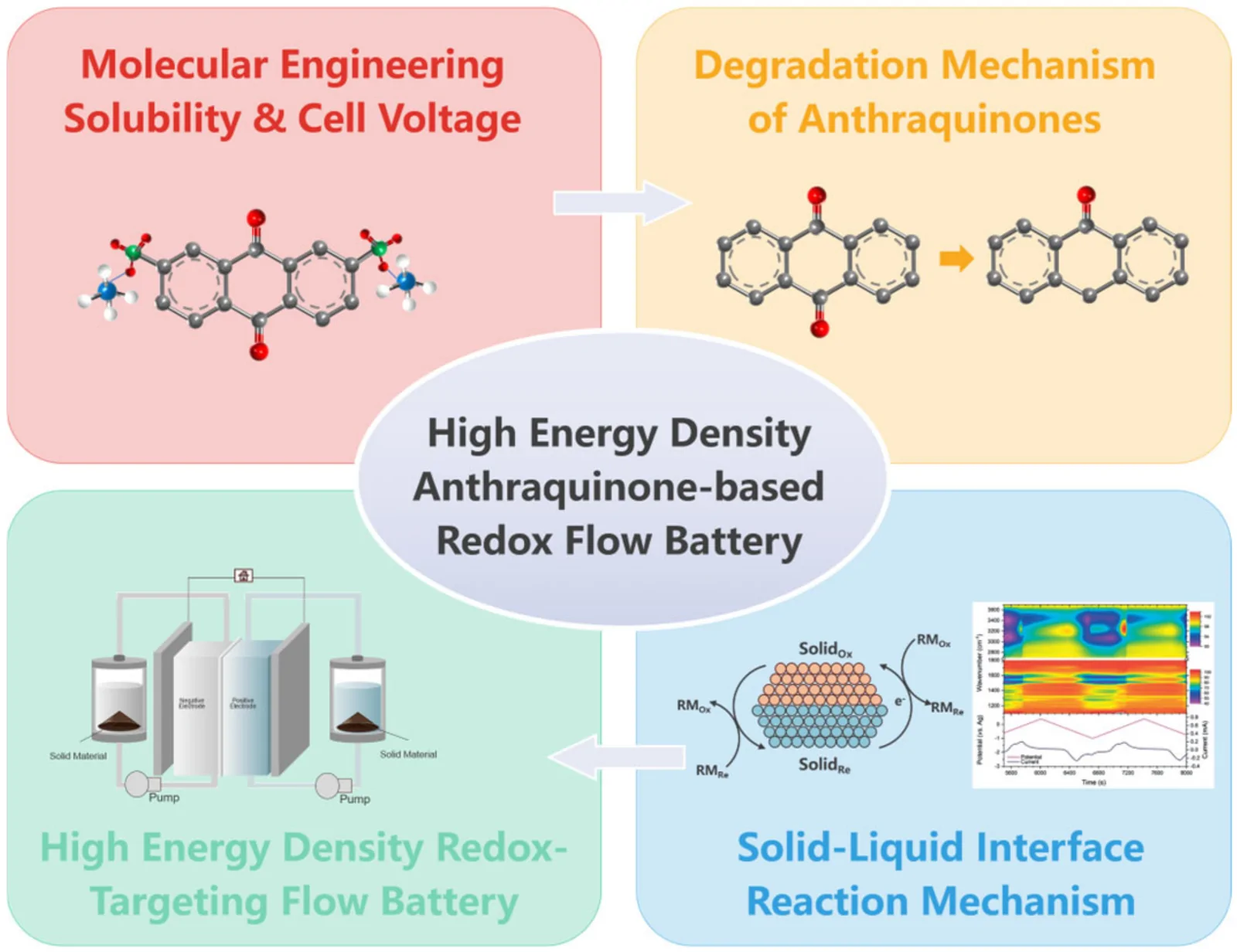
Schematic illustration of the research framework for high−energy−density anthraquinone−based redox flow batteries.

In this review, we summarize the development of anthraquinone-based redox flow batteries, emphasizing molecular engineering and redox-targeting systems for high energy density. The fundamental principles of anthraquinone redox chemistry and electron transfer mechanisms in the redox flow battery are firstly discussed. We then examine molecular design strategies for redox potential regulation, proton-coupled electron transfer kinetics, and aqueous solubility (Figure 1). Degradation pathways under concentrated solution cycling and molecular rejuvenation strategies are further assessed. The intrinsic solubility limit of homogeneous electrolytes motivates the shift to heterogeneous redox targeting systems for redox flow batteries. Recent advances in aqueous homogeneous electrolytes and solid/liquid redox-targeting systems are comprehensively analyzed. Key scientific issues include mediator and solid potential matching, interfacial charge transfer, solid active site utilization, and mass transport at reactor scale. The review summarizes in situ and ex situ characterization techniques to clarify redox-targeting processes, interfacial dynamics, and structural evolution. The last section of this review outlines current challenges and future perspectives, aiming to provide a solid basis for optimizing anthraquinone-based redox flow batteries and enabling practical high-energy-density storage.

## 2. Anthraquinone-Based Aqueous Redox Flow Batteries

### 2.1. Development of Anthraquinone-Based Aqueous Redox Flow Batteries

**Figure 2:**
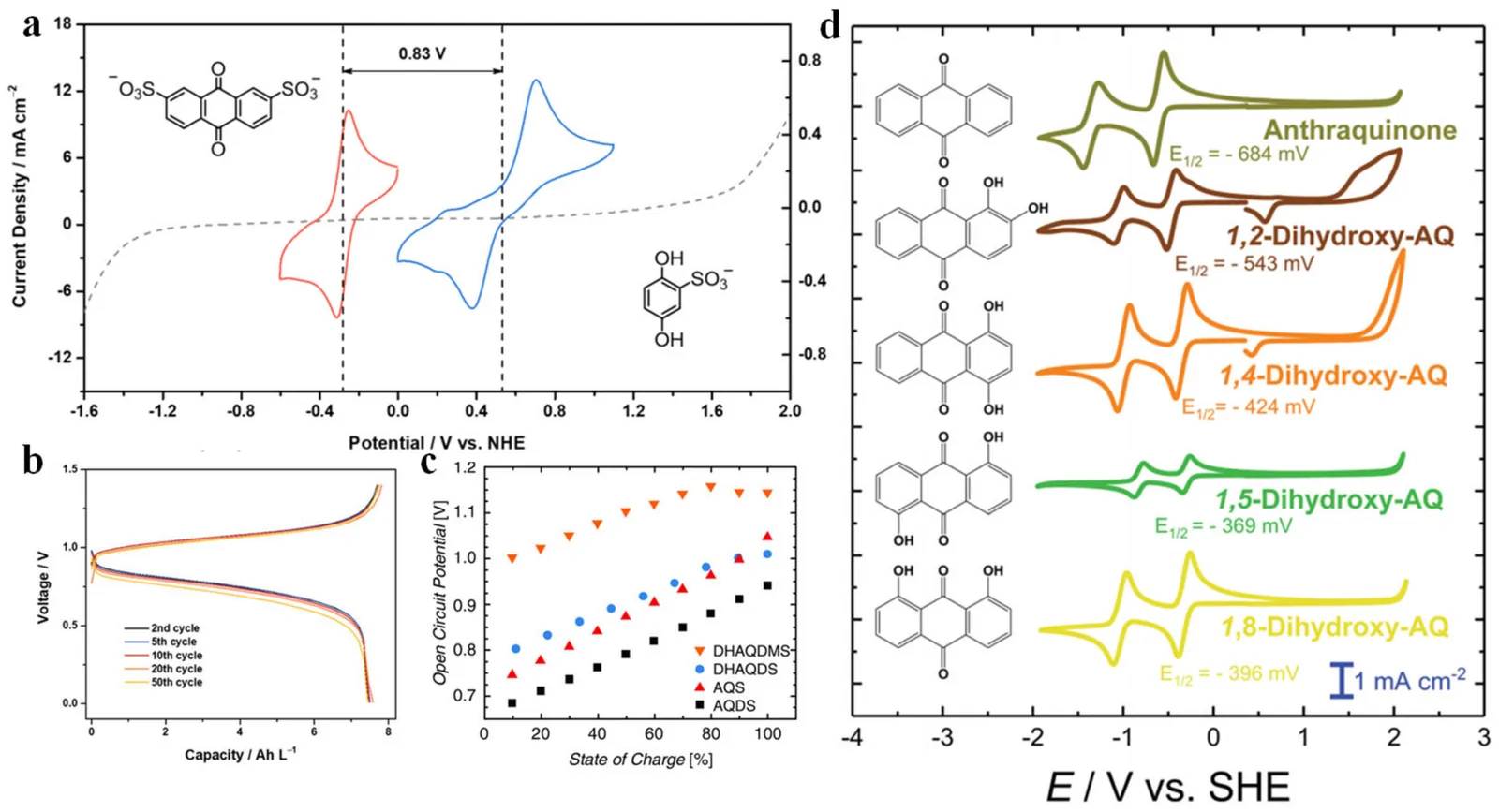
Electrochemical and molecular−design strategies for tuning anthraquinone redox properties. (**a**) Cyclic voltammogram of 2,7−AQDS and HQS (in 0.5 M Na_2_SO_4_), [43]. (**b**) Long−term cycling performance of the neutral all−quinone flow battery at 60 mA cm^−2^ [43]. (**c**) Comparison of the CV responses of AQDS, AQS, DHAQDS, and DHAQDMS [49]. (**d**) CV profiles of anthraquinone and its monosubstituted hydroxy− and amino−derivatives recorded in 0.1 M TBAPF_6_/DMF at a scan rate of 200 mV s^−1^ [57]. Reproduced with permission from the publisher [43,49,57].

In 2014, Huskinson et al. introduced an anthraquinone derivative into an aqueous flow battery, reporting a hybrid organic–inorganic system employing anthraquinone-2,7-disulfonic acid (AQDS) as the negative electrolyte and Br_2_/Br^−^ as the positive redox couple [41]. AQDS exhibits good aqueous solubility, and the carbonyl groups of the anthraquinone core undergo a reversible two-electron reduction. The significance of this work lies in demonstrating that anthraquinone molecules can function as reversible two-electron redox-active species in aqueous flow batteries, establishing the fundamental framework for anthraquinone-based aqueous organic RFBs. Lin et al. report an alkaline quinone flow battery employing 2,6-dihydroxyanthraquinone (2,6-DHAQ) as the negative active material and ferri/ferrocyanide as the positive redox couple [25]. This work marks a major transition from early acidic systems toward alkaline DHAQ-based aqueous flow batteries. Under alkaline conditions, the hydroxyl groups of DHAQ undergo deprotonation, modifying the electronic structure of the anthraquinone core and shifting its redox potential to more negative values; the alkaline environment can also improve the solubility of selected hydroxy-substituted anthraquinones. In subsequent work, the same group systematically elucidates the degradation mechanism of DHAQ during long-term cycling and [42], based on these findings, develop a series of strategies including pH regulation and state-of-charge control. Yang et al. develop an all-quinone neutral aqueous redox flow battery employing 2,7-AQDS and potassium 1,4-dihydroxybenzene sulfonate (HQS) as the negative and positive active materials (Figure 2a) [43]. The resulting battery demonstrates excellent cycling stability in a Na_2_SO_4_ electrolyte (Figure 2b). These approaches lead to an approximately 200-fold extension of the battery’s cycling lifetime, providing important mechanistic insights and practical guidance for improving the long-term stability of anthraquinone-based redox flow batteries. Research on anthraquinone flow batteries gradually shifted from demonstrating the performance of individual molecules toward molecular engineering.

Hydroxyl substitution can shift the redox potential to more negative values through its electron-donating effect, while also improving solubility in alkaline electrolytes through deprotonation of the hydroxyl groups. By contrast, sulfonate and carboxylate groups are commonly introduced to improve water compatibility, but their effects on redox potential depend on their electronic properties, substitution positions, and linkage to the anthraquinone core. For example, the position of sulfonate groups can alter both the redox potential and solubility because of differences in electronic effects, hydration, and crystal packing [44,45].

Side-chain engineering offers another way to balance solubility and electrochemical performance. The introduction of hydrophilic carboxylate-containing chains has produced anthraquinone electrolytes with high solubility and stable cycling. Wang et al. reported an N-alkyl-carboxylate-functionalized 2,6-diaminoanthraquinone derivative, N,N′-(9,10-anthraquinone-2,6-diyl)-di-β-alanine (DAEAQ), that delivered an open-circuit voltage of 1.12 V at pH ≥ 12 and a capacity retention rate of 99.86% per day under the reported cycling conditions [46]. Ether-linked side chains can also improve aqueous compatibility, but their position matters because ether-containing anthraquinones may undergo side-chain loss and anthrone formation during cycling. In a study of 2-2-propionate ether anthraquinone (2-2PEAQ), placing the ether linkages away from the central quinone ring reduced the capacity fade rate by approximately threefold relative to an isomer with linkages adjacent to that ring. Adding a neighboring amino group further suppressed the identified degradation pathways, yielding a derivative with a reported solubility of 1.1 M [47,48]. Gerhardt et al. systematically investigate a range of anthraquinone derivatives [49], showing that substituents on the anthraquinone core substantially modify redox potential, aqueous solubility, and chemical stability. These observations establish an important principle: the performance of an anthraquinone electrolyte is determined by the combined effects of molecular structure, electrolyte environment, and the counter redox couple. Optimizing a single parameter, such as solubility or redox potential, is therefore insufficient to obtain a practically attractive electrolyte.

Besides experimental developments of anthraquinone-based flow batteries, theoretical calculations are also utilized to support the screening of anthraquinone derivatives. Chen et al. use first-principles calculations to investigate how the position of hydroxyl substituents affects the redox potential and solubility of anthraquinone derivatives, identifying 1,8-DHAQ as a promising structure [50]. This work represents an early transition from empirical molecular screening toward computation-assisted molecular design of anthraquinone electrolytes. In 2018, Cao et al. systematically compared several DHAQ isomers, including 1,2-DHAQ, 1,4-DHAQ, 1,5-DHAQ, 1,8-DHAQ, and 2,6-DHAQ, in alkaline aqueous electrolytes [51]. Among the investigated structures, 1,8-DHAQ showed favorable electrochemical reversibility and mass-transfer characteristics, and its solubility could be enhanced using choline hydroxide as a solubilizing agent. As the voltage and power performance of anthraquinone flow batteries improved, long-term electrolyte stability and capacity decay became increasingly important limitations for practical applications. Kwabi et al. report an alkaline quinone flow battery with a long lifetime at approximately pH 12 by modifying the molecular structure of the quinone electrolyte and reducing the dependence on highly concentrated alkali [27]. Early studies mainly emphasized cell voltage, solubility, and power density of redox flow batteries. Afterwards, more advancements were developed with integrated optimization of voltage, solubility, reaction kinetics, and chemical stability. Meanwhile, the cost and scalability of active materials emerged as other considerations for the practical deployment of redox flow batteries. Wu et al. developed a high-performance anthraquinone derivative, 1,8-dihydroxy-2,7-dicarboxymethyl-9,10-anthraquinone (DCDHAQ), based on the 1,8-DHAQ framework and evaluated its properties from the perspectives of solubility, stability, synthesis, and cost [52]. Molecular design is increasingly assessed according to a broader set of requirements, encompassing not only electrochemical performance but also synthetic accessibility, scalable preparation, and economic viability. Beyond these molecular-level considerations, the cost and scalability of active materials merit expanded discussion, particularly regarding the trade-offs between molecular performance and synthetic accessibility. For instance, the low-cost sulfonated anthraquinone mixture strategy can lower material and purification costs by relaxing structural uniformity, although it may introduce additional trade-offs in redox potential, solubility, and cycling stability. By contrast, the cost-oriented molecular design of DCDHAQ seeks to preserve favorable redox properties while streamlining synthesis and reducing raw-material expenses. These examples underscore that molecular engineering must balance energy density, durability, and electrochemical reversibility against synthetic step count, yield, feedstock availability, purification burden, and process scalability. Consequently, the goal of molecular design has gradually shifted from identifying high-performing molecules at laboratory scale toward developing electrolyte materials capable of meeting the combined requirements of large-scale energy storage.

### 2.2. Molecular Engineering for Redox Potential Regulation of Anthraquinones

In anthraquinone-based redox flow batteries, a key molecular functionalization strategy is to increase the electron density of the anthraquinone core by introducing electron-donating substituents or extending the conjugated framework [53]. Such modifications can shift the redox potential toward more negative values, thereby increasing the overall cell voltage. Meanwhile, molecular functionalization must preserve or optimize the proton-transfer pathways associated with the carbonyl groups to maintain sufficiently rapid proton-coupled electron transfer (PCET) kinetics, preventing kinetic limitations from offsetting the thermodynamic benefits of potential tuning. A representative example is reported by Aziz et al. [41], who develop a metal-free redox flow battery based on the redox chemistry of 9,10-anthraquinone-2,7-disulfonic acid (AQDS). The Pourbaix diagram shows that the redox potential of AQDS, reported in mV versus the standard hydrogen electrode (SHE), is strongly pH-dependent. The fitted potential–pH relationship exhibits three distinct slopes of approximately −59, −30, and 0 mV pH^−1^, corresponding to potential shifts per unit increase in pH for two-electron redox processes involving the uptake of two, one, and zero protons, respectively (2e^−^/2H^+^, 2e^−^/1H^+^, and 2e^−^/0H^+^). Coupling the low-cost quinone/hydroquinone redox couple with the Br_2_/Br^−^ redox couple in sulfuric acid, the system delivers promising electrochemical performance, achieving a peak power density exceeding 0.6 W cm^−2^ at a current density of 1.3 A cm^−2^ while maintaining good cycling stability. The same study further demonstrates that molecular modification of AQDS could effectively tune its redox properties. Specifically, incorporation of two hydroxyl groups at the 1 and 8 positions of the anthraquinone framework increases the open-circuit voltage of the full cell by approximately 11%. Quantum-chemical calculations indicate that each hydroxyl substituent can lower the redox potential by approximately 50 mV, in good agreement with the experimental results, providing a broad potential-tuning range of nearly 0.6 V. In addition, hydrogen-bonding interactions associated with hydroxyl substitution are expected to enhance aqueous solubility, while intramolecular hydrogen bonding between hydroxyl and carbonyl groups can facilitate the associated redox kinetics. This work establishes molecular functionalization as an effective approach for tuning the redox potential of quinone-based electrolytes and increasing the operating voltage of aqueous redox flow batteries.

For proton-coupled electron-transfer processes, the redox potential of anthraquinone derivatives generally shifts linearly toward more negative values as the pH increases, consistent with the Nernst equation. In an ideal two-proton/two-electron process, the potential decreases by approximately 59 mV for every unit increase in pH. Taking advantage of this pH-dependent redox behavior, Lin and co-workers develop a high-voltage anthraquinone-based aqueous redox flow battery operating under alkaline conditions [25]. The selected 2,6-dihydroxyanthraquinone (2,6-DHAQ) exhibited a room-temperature solubility exceeding 0.6 M in 1 M KOH and showed a reversible redox process near −0.7 V versus the standard hydrogen electrode (SHE). When paired with the ferrocyanide/ferricyanide redox couple, the system achieves a cell voltage of approximately 1.2 V. The relatively high operating voltage of the alkaline system also helps compensate for the increased membrane resistance, enabling electrochemical performance and kinetics comparable to those of conventional all-vanadium redox flow batteries. The battery delivers a peak power density above 0.45 W cm^−2^ at room temperature and approximately 0.7 W cm^−2^ at 45 °C. At a high current density of 100 mA cm^−2^, an energy efficiency of 84% was achieved, while the capacity retention exceeded 99.9% per cycle. These results highlight the potential of hydroxyl-functionalized anthraquinones and ferrocyanide-based redox couples as stable electrolytes for high-performance alkaline aqueous redox flow batteries. Michael and co-workers further investigate four anthraquinone derivatives as candidate negative electrodes for aqueous quinone–bromine redox flow batteries [49], including anthraquinone-2-sulfonic acid (AQS), 1,8-dihydroxyanthraquinone-2,7-disulfonic acid (DHAQDS), alizarin red (ARS), and 1,4-dihydroxyanthraquinone-2,3-dimethylsulfonic acid (DHAQDMS). All four derivatives exhibit more negative standard reduction potentials than anthraquinone-2,7-disulfonic acid (AQDS), thereby providing an opportunity to increase the open-circuit voltage. The lower redox potential of AQS relative to AQDS was attributed to the removal of one electron-withdrawing sulfonic acid group, whereas the introduction of electron-donating hydroxyl groups further shifted the reduction potential of DHAQDS toward negative values (Figure 2c). The DHAQDMS results further suggest that increasing the spatial separation between electron-withdrawing substituents and the redox-active core can provide an additional means of lowering the quinone reduction potential. However, both DHAQDS and DHAQDMS exhibit a decrease in open-circuit voltage at high state of charge (higher than 80% SOC), which was attributed to chemical degradation reactions occurring during operation. These degradation pathways ultimately limited the practical applicability of these highly reduced-potential quinone derivatives in redox flow batteries.

### 2.3. Computational Studies on the Thermodynamics of Anthraquinones

Beyond anthraquinones, the redox potentials of quinones and other carbonyl-containing redox polymers can also be tuned through the introduction of electron-donating or electron-withdrawing substituents [54,55]. Density functional theory (DFT) calculations are employed to systematically investigate the electrochemical properties of a wide range of anthraquinone (AQ) derivatives [56], including the redox window, the effects of electron-donating and electron-withdrawing substituents on the redox window, and solvation free energies, establishing a correlation between the calculated LUMO energy levels and reduction potentials. An expression was further derived to predict the reduction potentials of anthraquinone derivatives from their calculated LUMO energy levels. This equation can serve as a descriptor for evaluating the reduction potentials of thousands of anthraquinone derivatives without requiring detailed free-energy calculations for the neutral and reduced electronic states. These findings provide an ideal platform for combined experimental and theoretical studies aimed at understanding the electrochemical reversibility and solubility of novel quinone-based molecules as energy storage materials. Because anthraquinones offer a large number of possible substitution patterns, whereas commercially available anthraquinone derivatives remain relatively limited, Josef et al. [57] systematically evaluated several semi-empirical density-functional tight-binding (DFTB) approaches for predicting and screening the electrochemical properties of anthraquinone derivatives. The cyclic voltammograms of pristine anthraquinone (AQ) and its monosubstituted derivatives revealed two distinct redox couples (Figure 2d), indicating that these derivatives retained electrochemical activity and generally exhibited reversible redox behavior. Amino substitution, owing to its electron-donating effect, shifted the redox potential toward more negative values. Although hydroxyl substitution is also expected to increase the electron density of the anthraquinone framework, the 2-OH-AQ derivative represented a particularly challenging case in virtual screening because of the possible stabilization of radical intermediates through intramolecular interactions and/or dimerization reactions. This observation demonstrates that single-molecule quantum-chemical calculations may not fully capture intermolecular processes, such as dimerization and other side reactions, that can influence experimentally measured redox properties.

High-throughput virtual screening (HTVS) can further extend this approach to thousands of candidate structures by combining quantum-chemical calculations with ML models trained on computed descriptors, molecular structures, or molecular representations. Such workflows can rapidly evaluate and prioritize anthraquinone and related quinone derivatives according to their redox potentials and solubility, while incorporating separate computational or ML-based filters for molecular stability and susceptibility to competing reactions. For example, a data-driven screening study examined 3257 quinone-based redox pairs and combined quantum-chemical redox-property calculations with ML-based aqueous-solubility prediction, identifying 205 candidates predicted to offer both higher solubility and lower redox potentials than anthraquinone-2,7-disulfonic acid (AQDS) [58]. In another study, DFT-generated data for more than 8200 quinone-type molecules were used to train ML models for proton-coupled electron-transfer properties, yielding a mean absolute error of approximately 66 mV for redox-potential prediction [59]. Molecular distortion and the energetics of competing chemical reactions can provide additional stability-related screening criteria [60]. More broadly, ML surrogates trained on quantum-chemical calculations have also been employed to accelerate the discovery of stable organic redox-active molecules [61].

Solvation and explicit-environment modeling are equally important for improving the reliability of predicted redox potentials. Implicit solvation models, such as COSMO and the polarizable continuum model (PCM), provide an efficient approximation of bulk solvent effects but may not fully describe specific hydrogen bonding, counterion association, or local solvent-mediated reactions. For anthraquinone derivatives, DFT calculations have indicated that lithium-ion pairing can shift the reduction potential by approximately 0.4 V [56]. In a separate combined computational and experimental study, continuum-solvation calculations produced substantially larger errors for certain anthraquinone derivatives because intramolecular hydrogen bonding was not adequately balanced against hydrogen bonding with the surrounding solvent; incorporating explicit water molecules through quantum mechanics/molecular mechanics (QM/MM) simulations improved the predictions for these cases [62]. Further computational screening of quinone derivatives has shown that intramolecular hydrogen bonding, conformational effects, and explicit water molecules can also modify predicted redox potentials and reveal possible competing reactions [60].

Nevertheless, the calculated values for most derivatives showed good agreement with experimental measurements. The SCC-DFTB/3ob method combined with the COSMO solvation model therefore provides a useful computational framework for screening candidate structures, which can subsequently be evaluated using higher-level DFT calculations to systematically identify high-performance organic redox-active molecules.

Regulating the potential of the active electrode materials in flow batteries to enhance the full-cell voltage is one of the important strategies for improving flow battery performance. However, molecules with favorable redox thermodynamics may nevertheless suffer from poor aqueous solubility because of strong aromatic π-π stacking interactions and high lattice energies. Insufficient solubility limits the concentration of electroactive species and therefore constrains the achievable energy density. Furthermore, precipitation of poorly soluble active materials during charging can lead to irreversible deposition on the electrode surface and accelerate capacity decay. Consequently, molecular design must simultaneously balance redox-potential tuning and aqueous solubility rather than optimizing either property independently.

## 3. Hydrophilic Functionalization and Solubility Enhancement of Anthraquinones

### 3.1. Cation Regulation for Enhanced Anthraquinone Solubility

**Figure 3:**
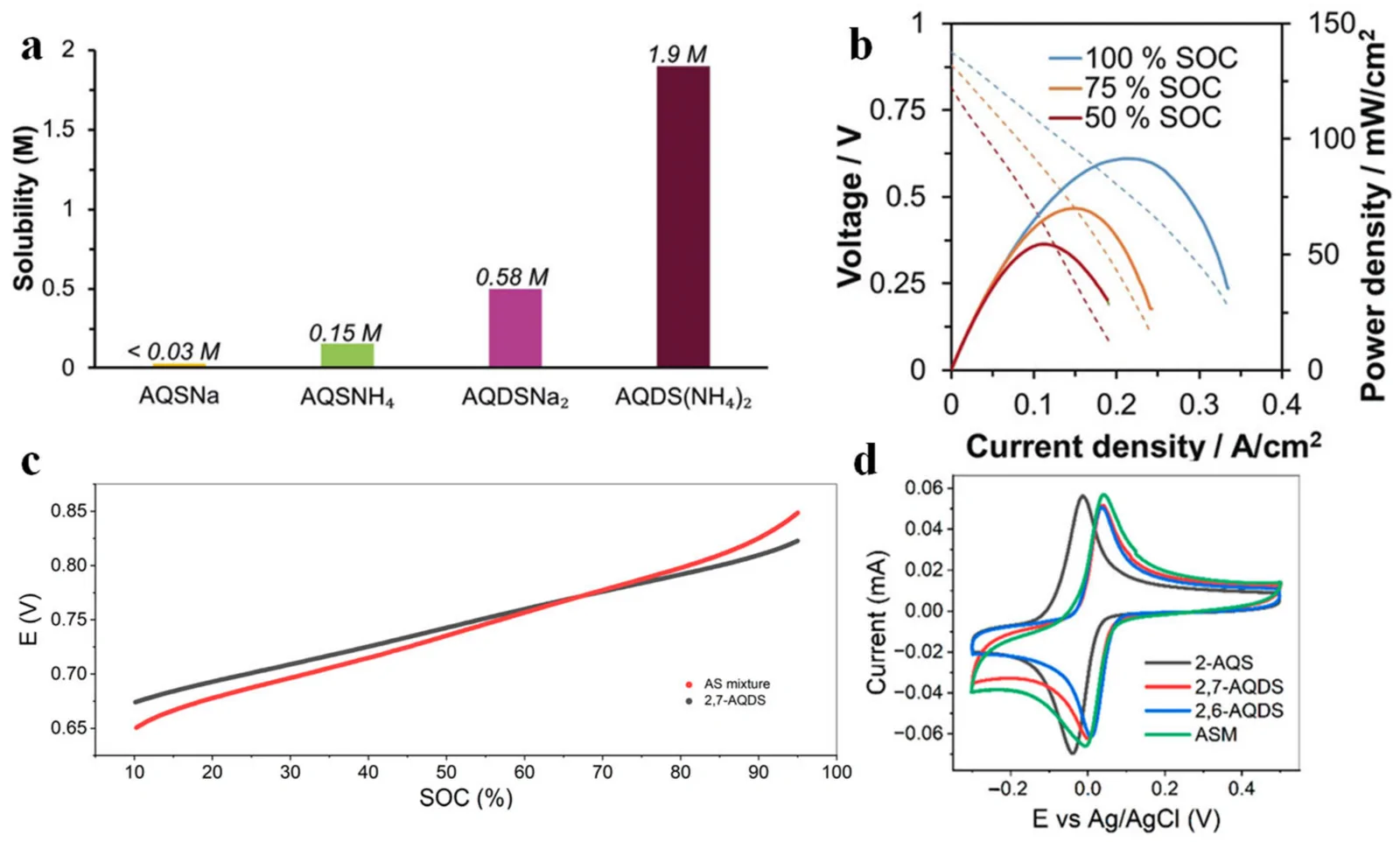
Molecular and electrolyte engineering approaches for enhancing anthraquinone solubility and battery performance. (**a**) Comparison of the aqueous solubilities of sodium- and ammonium-based AQS and AQDS salts [64]. (**b**) Cell power density as a function of state of charge [64]. (**c**) The OCV–SOC relationship of flow batteries using ASM or 2,7-AQDS (0.4 M in 2.2 M H_2_SO_4_) as the negative electrolyte [67]. (**d**) CV responses of the individual sulfonated anthraquinones and their mixed-electrolyte formulation in 1 M H_2_SO_4_ at a scan rate of 50 mV s^−1^ [67]. Reproduced with permission from the publisher [64,67].

Beyond redox-potential tuning, improving the solubility of redox-active species represents another key strategy for increasing the energy density of aqueous organic redox-flow batteries [63]. Accordingly, extensive efforts have focused on molecular and electrolyte engineering to achieve higher active-material concentrations. One representative strategy for improving solubility is counterion engineering. For example [64], replacing Na^+^ with NH_4_^+^ as the counterion of AQDS produces (NH_4_)_2_AQDS, whose aqueous solubility can reach approximately 1.9 M (Figure 3a), and finally shows a higher power density of 91.5 mW cm^−2^ (Figure 3b) at 100 % SOC. This enhancement has been attributed to the strong hydrophilicity of NH_4_^+^ and hydrogen-bonding interactions between NH_4_^+^ and AQDS^2−^, which facilitate hydration and weaken crystal packing. This approach substantially increases the concentration of electroactive species while maintaining excellent cycling stability, with nearly no capacity decay over 300 cycles. Interestingly, the redox potential was measured across a range of pH values to construct its Pourbaix diagram, which revealed that the redox potential remains nearly constant between pH 4 and 7, indicating a two-electron transfer process that is decoupled from proton transfer within this pH range. This phenomenon is attributed to hydrogen-bonding interactions between the ammonium cations and the carbonyl oxygen atoms of the AQDS anion. Choline cations act as solubilizing agents by forming interionic hydrogen bonds with quinone sulfonate anions, thereby enabling novel active organic electrolytes with high aqueous solubility, good electrochemical reversibility, and excellent stability. Typically, choline anthraquinone sulfonate (AQSCH) and dicholine 1,2-dihydroxy-3,5-phenylenedisulfonate (BQDS(CH)) are synthesized through simple ion-exchange and acid-base neutralization reactions, which can be employed as negative and positive electrolytes in pH-neutral aqueous organic redox flow batteries (AORFBs) [65]. Owing to the weakened ionic interactions and the formation of hydrogen bonds between cations and anions, the aqueous solubility of anthraquinone and benzoquinone derivatives is enhanced by up to tens of times.

### 3.2. Molecular Substituent Regulation for Improved Aqueous Solubility of Anthraquinones

In addition to counterion engineering, molecular functionalization provides an effective strategy for increasing the aqueous solubility of anthraquinone derivatives. Kerr et al. reported 2,2′-((9,10-dioxo-9,10-dihydroanthracene-2,6-diyl)bis(oxy))dipropionic acid (2,6-D2PEAQ), an anthraquinone derivative incorporating ether linkages and chiral, methyl-branched side chains terminated with carboxylic acid groups [66]. At approximately neutral pH, the compound exhibits a high aqueous solubility of up to approximately 2.0 M, corresponding to a theoretical negative-electrolyte volumetric capacity of 107.2 Ah/L for a two-electron redox process. Its measured reduction potential is −0.460 V vs. SHE at pH 7. Under practical cell-testing conditions, a nearly capacity-balanced cell employing 1.1 M 2,6-D2PEAQ and a ferro-/ferricyanide-based positive electrolyte exhibited an open-circuit voltage of 1.09 V at 90% state of charge (SOC). The reported realized volumetric capacity was 59.0 Ah/L based on the negative-electrolyte volume, while the volumetric capacity based on the total battery electrolyte volume was 14.3 Ah/L; the corresponding reported cell energy density was 13.6 Wh/L. The cell demonstrated a capacity-fade rate of 0.02% per day over 14 days, with a coulombic efficiency of 99.7% [66]. The solubility of 2,6-D2PEAQ is also influenced by counterion identity: reported measurements indicate that the potassium salt can reach approximately 1.9–2.0 M at pH 7, compared with approximately 1.5 M for its sodium analogue. Accordingly, its compatibility with mixed Na^+^/K^+^ electrolyte formulations should not be interpreted as evidence of completely counterion-independent solubility.

Another effective molecular-functionalization strategy involves introducing polyethylene glycol (PEG)-based solubilizing groups. Jin et al. synthesized and screened a series of PEG-substituted anthraquinones (PEGAQs) and selected 1,8-bis(2-(2-(2-hydroxyethoxy)ethoxy)ethoxy)anthracene-9,10-dione (AQ-1,8-3E-OH) for detailed electrochemical evaluation [30]. This derivative is water-miscible over a broad pH range, enabling the preparation of concentrated aqueous electrolytes without relying on ionizable solubilizing groups. Its reported reduction potential is −0.43 V vs. SHE at pH 7. At an operating concentration of 1.5 M, the negative electrolyte delivers a reported volumetric capacity of 80.4 Ah/L. When paired with a ferrocyanide-based positive electrolyte at pH 7, the cell exhibits an open-circuit voltage of approximately 1.0 V and a reported energy density of 25.2 Wh/L. Cycling experiments conducted for 220 cycles over 18 days yielded an average coulombic efficiency of 99.90%, with a capacity-fade rate of 0.5% per day, equivalent to 0.043% per cycle [28]. Although AQ-1,8-3E-OH provides a higher reported operating volumetric capacity and cell energy density under the respective reported conditions, its measured capacity-fade rate is higher than that of 2,6-D2PEAQ.

Similarly, David and co-workers functionalize 2,6-DHAQ with highly alkaline-soluble carboxylate termini [27]. The solubilizing groups are connected to the anthraquinone core through relatively short hydrophobic linkers, which increase solubility by disrupting crystal packing while avoiding excessive hydrophobicity. Meanwhile, cyclic voltammetry shows that all 4,4′-((9,10-anthraquinone-2,6-diyl)-dioxy)-dibutyrate (DBEAQ) isomers exhibit very similar redox potentials. The resulting derivative, 2,6-DBEAQ, shows a solubility approximately six times higher than that of 2,6-DHAQ in a pH 12 supporting electrolyte, enabling a full-cell energy density of approximately 17 Wh L^−1^. Petrov et al. demonstrate another low-cost strategy based on blending sulfonated anthraquinone isomers to exploit a eutectic-like solubility enhancement [67]. Sulfonation of anthraquinone with fuming sulfuric acid produced mixtures containing several sulfonated derivatives, including 2,7-AQDS, 2,6-AQDS, and AQS, together with unreacted anthraquinone dissolved in diluted sulfuric acid. Combined with optimization of the membrane and flow-field configuration, the resulting redox flow battery achieves a maximum power density of 398 mW cm^−2^ at 100% SOC. The OCV–SOC relationship (Figure 3c) of flow batteries using ASM or 2,7-AQDS (0.4 M in 2.2 M H_2_SO_4_) as the negative electrolyte also supports the increase in power density. The resulting mixed-electrolyte system substantially increases the concentration of active quinone species without significantly altering their fundamental electrochemical characteristics (Figure 3d). Overall, increasing the solubility of redox-active species is an effective strategy for enhancing the energy density of aqueous organic redox-flow batteries. Molecular functionalization, counterion engineering, and modulation of intermolecular interactions can suppress π–π stacking and improve the achievable active-material concentration. However, excessively high concentrations may also accelerate the accumulation of side products during long-term cycling, potentially aggravating active-material loss and capacity decay. Therefore, achieving a balance between high solubility and long-term chemical stability remains a critical challenge for practical high-energy-density organic flow batteries.

## 4. Degradation Mechanisms and Molecular Rejuvenation of Anthraquinone Electrolytes

### 4.1. Degradation Pathways of Anthraquinone Electrolytes

**Figure 4:**
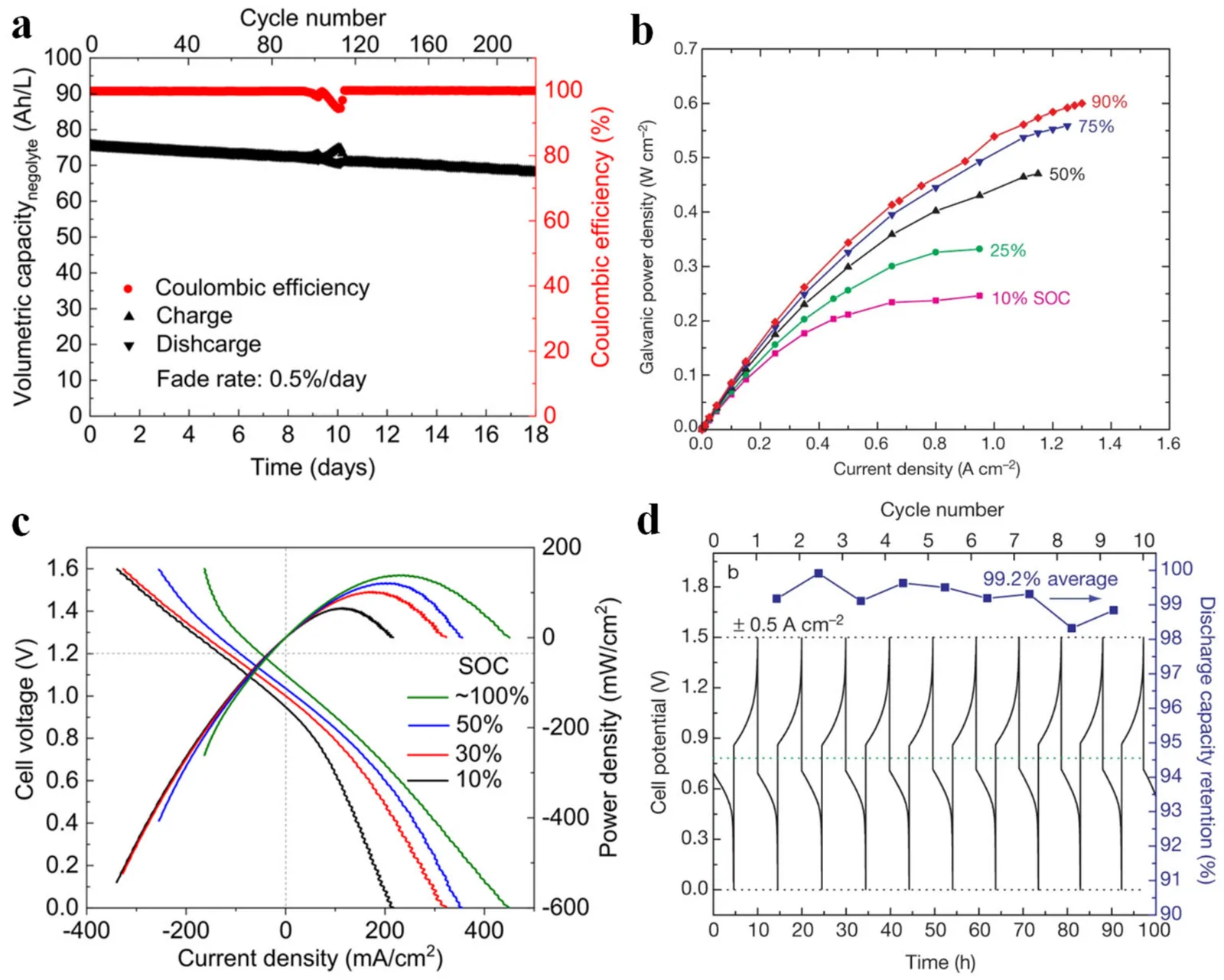
Degradation, diagnostics, and rejuvenation in anthraquinone flow batteries. (**a**) The relationship between coulombic efficiency and time [83]. (**b**) The relationship between power density and current density at five distinct SOCs with a solution containing 3 M HBr and 0.5 M Br_2_ on the cathodic side, and a combination of 1 M AQDS and 1 M H_2_SO_4_ on the anodic side [83]. (**c**) Voltage profiles versus current density at ambient temperature at four distinct SOCs. The electrolyte composition consists of 7 mL of 1.5 M PEGAQ [83]. (**d**) Discharge capacity retention and cell potential versus cycle number [83]. Reproduced with permission from the publisher [83].

The central challenge confronting anthraquinone-based aqueous organic redox flow batteries under deep cycling is that reduced anthrahydroquinones do not represent thermodynamic end states [68]. They instead lose electrochemical activity through multiple chemical pathways. Mapping these microscopic degradation channels provides the logical starting point for designing long-lived electrolytes. The most systematically studied case is 2,6-dihydroxyanthraquinone in alkaline media. Its canonical reduction proceeds as a two-electron, two-proton transfer that yields the dihydroxyanthrahydroquinone dianion. At high states of charge, however, DHAHQ^2−^ undergoes deprotonation followed by tautomerization into the more reactive anthrone intermediate, 2,6-dihydroxyanthrone. A lower-energy aromatic conjugated system drives this tautomerization, yet the quinonoid scaffold forfeits its reversible redox activity in the process. High-resolution mass spectrometry and Nuclear Magnetic Resonance (NMR) Spectroscopy confirm that DHA is the first detectable decomposition product in DHAQ electrolytes, and its accumulation scales quantitatively with capacity fade [31]. The life of this anthrone intermediate sets the decay rate. Under alkaline conditions, DHA deprotonates further to an anthrone anion radical. Electrochemical dimerization then couples two radicals through a C-C bond, yielding the electrochemically inert bisanthrone dimer. This process is irreversible, and each dimerization event permanently removes active material. Quantitative analysis attributes over 95% of the roughly 4.3% per day fade in standard DHAQ/ferricyanide full cells to anthrone formation and subsequent dimerization. A representative DHAQ/ferricyanide flow cell exhibits a volumetric capacity fade of 0.5% per day over 200 cycles (Figure 4a), a decay that the following mechanistic analysis attributes primarily to anthrone formation and irreversible dimerization [28].

Tautomerization-driven degradation is not unique to anthraquinones. Investigating the natural naphthoquinone derivative 2-hydroxy-1,4-naphthoquinone, Zhang et al. find that its reduced form likewise favors keto-enol tautomerization with a DFT-calculated barrier of only 2.42 kJ mol^−1^, leading to severe capacity fade. Grafting a benzoic acid side chain to construct the nonplanar π-extended BANQ raises this barrier to 4.98 kJ mol^−1^ and places the tautomerized product higher in energy than reduced BANQ itself, thermodynamically closing the tautomerization channel and suppressing the fade rate to 0.00018% per cycle [69]. This finding extends the tautomerization-driven degradation paradigm to the broader quinone family and suggests π-extension and nonplanar conformation as general suppression strategies.

Under strongly alkaline conditions, hydroxide-mediated nucleophilic attack can induce substitution and cleavage of ether-linked side chains from anthraquinone derivatives [45]. Carboxylate- and other side-chain-functionalized anthraquinones can also undergo side-chain loss or decarboxylation under alkaline operating conditions, depending on molecular structure [47]. In the fused quinone-pyrazine molecule 2,2′-((6,11-dioxo-6,11-dihydrobenzo- [b]-phenazine-2,3-diyl)-bis-(oxy))-dipropionic acid (DCNQBP), Xu et al. trace degradation to hydrolysis-induced cleavage of propionic acid side chains rather than quinone-core instability, elevating side-chain stability from an empirical consideration to a quantitatively designable parameter [70]. Dissolved oxygen further accelerates chemical oxidation of reduced species, generating superoxide radicals that promote polymerization and cross-linking. These side reactions become particularly pronounced at high concentration, high state of charge, and elevated temperature. Molecular engineering offers a complementary route to source-level suppression. Fusing anthraquinone with phenazine, Zhang et al. construct 4,4′-((6,11-dioxo-6,11-dihydrobenzo- [b]-phenazine-2,3-diyl)-bis-(oxy))-dibutanoic acid (DAPQ), a four-electron storage molecule whose extended π-conjugation and balanced charge distribution act in concert to suppress anthraquinone-characteristic disproportionation and phenazine-characteristic dimerization. In situ NMR detects no degradation products after 2300 cycles, with a fade rate of 0.0017% per day [71]. Approaching from natural pharmaceutical anthraquinones, Ning et al. find that intramolecular and NH_4_Cl-induced intermolecular hydrogen bonds stabilize the reduced state, cutting the fade rate of rhein-based cells from 0.124% to 0.011% per cycle [72]. Collectively, these studies show that degradation-mechanism elucidation is now driving molecular design in reverse, shifting the paradigm from trial-and-error screening to precision suppression.

### 4.2. Operando Diagnostics and Computational Validation of Anthraquinone Degradation Mechanism

Elucidating the anthraquinone degradation mechanism requires the synergy of advanced characterization and theoretical computation. Conventional ex situ analyses require electrolyte extraction, a procedure that can itself alter the chemical state of reduced species, making in situ and operando techniques essential for real-time degradation tracking [73]. Operando NMR has therefore emerged as a particularly powerful diagnostic approach for tracking anthraquinone degradation in real time [74,75], with studies demonstrating quantitative monitoring of DHAQ decomposition and the formation of 2,6-dihydroxyanthrone and 2,6-dihydroxyanthranol. Zhao et al. design a flow NMR probe coupled to a redox flow cell and acquire ^1^H NMR spectra during cycling of a 100 mM DHAQ/ferricyanide full cell. The pseudo-two-dimensional spectra reveal a clear sequence: early in charging, DHAQ^2−^ aromatic proton peaks diminish while intermediate DHAQ^3−^ peaks emerge and broaden; at deep charge, the semiquinone DHAQ•^−^ signal undergoes dramatic line broadening and an upfield shift due to paramagnetism. DFT-calculated volumetric shifts enable unambiguous assignment of all three states. More critically, holding the cell at 1.7 V produces new resonances assignable to DHA and DHAL, marking the first in situ observation of degradation-product formation in a functioning battery [76].

Electron paramagnetic resonance complements NMR by capturing semiquinone radical transients. In a follow-up study combining NMR with Electron Paramagnetic Resonance (EPR), Zhao et al. measure an intermolecular electron exchange rate constant k_ex_ of approximately 10^4^ M^−1^s^−1^ between DHAQ^2−^ and DHAQ•^−^, a parameter that directly governs the probability of anthrone-generating disproportionation [77]. In newer systems such as DAPQ, EPR reveals that the radical signal peaks at 50% state of charge and decays through rapid electron exchange, evidence that its flip-flop spin-exchange mechanism favors reversible conversion over side reactions [71]. At the electrochemical impedance level, distribution of relaxation times analysis offers a powerful tool for deconvolving interfacial transport from charge-transfer polarization. The SOC-dependent polarization curves of DHAQ/ferricyanide cells (Figure 4b,c) exhibit distinct ohmic, activation, and mass-transport regimes, which DRT analysis can deconvolve into ohmic, charge-transfer, and concentration-diffusion components to distinguish membrane-fouling-induced transport fade from molecular-degradation-induced charge-transfer fade [28,41]. Conventional Electrochemical Impedance Spectroscopy (EIS) yields only a total-impedance Nyquist plot, whereas Distribution of Relaxation Times (DRT) applies regularized deconvolution to resolve multiple RC elements with characteristic time constants corresponding to ohmic, distributed-ohmic, charge-transfer, and concentration-diffusion resistances [78,79]. In a bislawsone/polysulfide system, Chen et al. couple in situ DRT with Raman spectroscopy and identify the decrease in charge-transfer resistance as a direct signature of the bislawsone catalytic effect [80]. This methodology transfers directly to anthraquinone systems, providing a quantitative criterion for distinguishing membrane-fouling-induced transport fade from molecular-degradation-induced charge-transfer fade.

Theoretical computation supplies an atomistic rationale. Hashemi et al. develop a DFT-plus-machine-learning method for predicting quinone square schemes, decomposing a two-proton, two-electron transfer into eight elementary electron-transfer and eight proton-transfer steps. The E° and pKa of each step are computed independently by DFT, yielding a complete Pourbaix diagram [59]. For 2,2PEAQ as a benchmark, the predicted diagram agrees closely with experiment, delineating a 2e^−^/2H^+^ region at pH 0–5, a 2e^−^/1H^+^ transition at pH 5–8, and a 2e^−^/0H^+^ plateau above pH 8. This framework predicts the thermodynamic stability of every protonation and oxidation state at arbitrary pH, identifying when a reduced species enters an instability zone that triggers tautomerization. DFT transition-state calculations go further by quantifying tautomerization and dimerization free-energy barriers, as exemplified by the BANQ study’s barrier increase from 2.42 to 4.98 kJ mol^−1^, which provides molecular design with a quantitative degradation-propensity metric [69].

### 4.3. Mechanism-Guided Rejuvenation Strategies for Anthraquinone Electrolytes

A deeper understanding of degradation mechanisms has spawned a suite of mechanism-guided in situ rejuvenation strategies whose shared principle is intervention at distinct nodes of the degradation chain to reverse or slow capacity fade [81]. The first strategy defines state-of-charge operating boundaries that bypass over-reduction zones at the source. Because anthrone formation requires DHAHQ^2−^ deprotonation and tautomerization at high state of charge, limiting the charge cutoff voltage suppresses DHA generation substantially. Goulet et al. demonstrate that lowering the cutoff of a DHAQ/ferricyanide full cell from 1.6 V to 1.25 V collapses the fade rate from 5.6% to 0.14% per day, a roughly 40-fold lifetime extension [31]. Ning et al. independently validate this strategy in a natural anthraquinone system, where restricting rhein-based cells to 70% state of charge combined with NH_4_Cl hydrogen-bond stabilization lowers the fade rate to 1.85% per day [72]. Xu et al. make a parallel observation in DCNQBP, where avoiding deep discharge mitigates hydrolysis-induced side-chain cleavage and holds the fade rate at 0.018% per day [70]. The consistency of these results across chemically distinct systems establishes state-of-charge boundaries as the most universal and lowest-cost rejuvenation method. Another strategy establishes electrochemical pulse protocols that actively cleave C-C bonds in preformed dimers. Bahari et al. systematically investigate DHAQ electrolyte rejuvenation conditions and find that applying a higher discharge-step cutoff voltage not only oxidizes DHA back to DHAQ^2−^ but also further oxidizes the dimer, breaking its C-C bonds and regenerating active monomers. The voltage-time profile resolves three stages: DHA oxidation, dimer oxidation, and normal cycling of regenerated DHAQ. Combined with OH^−^ concentration optimization, precise cutoff-voltage tuning, and optimized periodic rejuvenation intervals, this strategy reduces the fade rate from 4.3% to 0.02% per day, a roughly 200-fold lifetime extension [42]. Rejuvenation efficiency remains highly parameter-sensitive, however, as insufficient voltage fails to cleave the dimer while excessive voltage triggers oxygen evolution and pH drift, making real-time DRT-EIS feedback indispensable. Whereas standard cycling at ±0.5 A cm^−2^ yields well-defined voltage plateaus but gradual capacity loss (Figure 4d), inserting a higher-voltage oxidation pulse during the discharge step cleaves preformed dimer C–C bonds and recovers active monomers, reducing the fade rate from 4.3% to 0.02% per day [41]. A third strategy combines controlled chemical oxidation with mixed-electrolyte synergistic rebalancing. Mild aeration represents the simplest approach: bubbling a small amount of air through a faded anolyte exploits molecular oxygen to chemically oxidize DHA and dimers back to DHAQ. In symmetric-cell tests, Goulet et al. show that aerating a DHAQ electrolyte cycled to 35% of its initial capacity restores discharge capacity to approximately 70% [31], though the aeration dose requires precise control to avoid over-oxidation.

The mixed-electrolyte strategy represents a more nuanced design. Lee et al. find that introducing DBEAQ, which carries a more positive reduction potential than DHAQ, continuously catalyzes degradation-product regeneration during normal cycling. Operating as a redox shuttle, reduced DBEAQ cleaves dimers into DHA during discharge, while oxidized DBEAQ oxidizes DHA back to DHAQ during charge, continuously scavenging degradation intermediates. A 0.1 M DHAQ plus 0.1 M DBEAQ mixture fades at only 0.1% per day, far below the 2.35% per day expected for a noninteracting mixture, and this gap demonstrates a pronounced synergistic effect [82]. The strategy’s generality has been verified across N-TSAQ and DPPEAQ derivatives, establishing a new paradigm for self-healing electrolytes. These rejuvenation strategies and molecular structure design are not mutually exclusive but can be layered for a combined effect. DAPQ suppresses disproportionation and dimerization at the source through its fused structure, BANQ raises the tautomerization barrier through nonplanar π-conjugation, and combining either approach with state-of-charge boundary control and electrochemical rejuvenation brings near-zero-fade operation within reach.

## 5. Anthraquinone-Based Redox-Targeting Flow Batteries

### 5.1. Concepts of Redox-Targeting Reaction

Diagnostic techniques and molecular rejuvenation strategies have extended anthraquinone electrolyte lifetimes substantially [84]. Homogeneous flow batteries nevertheless confront an inescapable physical constraint: the solubility limit of liquid solvents [85]. In anthraquinone-based systems, aggregation and limited aqueous solubility can consequently restrict the attainable volumetric energy density. Highly concentrated electrolytes can approach the molar scale, but further increases require additional molecular or solvation engineering; for example, a 2025 study achieved 1.6 M and 74.3 Ah L^−1^ using solvation-structure modulation [85]. At the same time, recent multi-electron molecular designs demonstrate that volumetric capacities can exceed 100 Ah L^−1^, indicating that 100 Ah L^−1^ should be regarded as a practical benchmark rather than an absolute physical limit [86].

**Figure 5:**
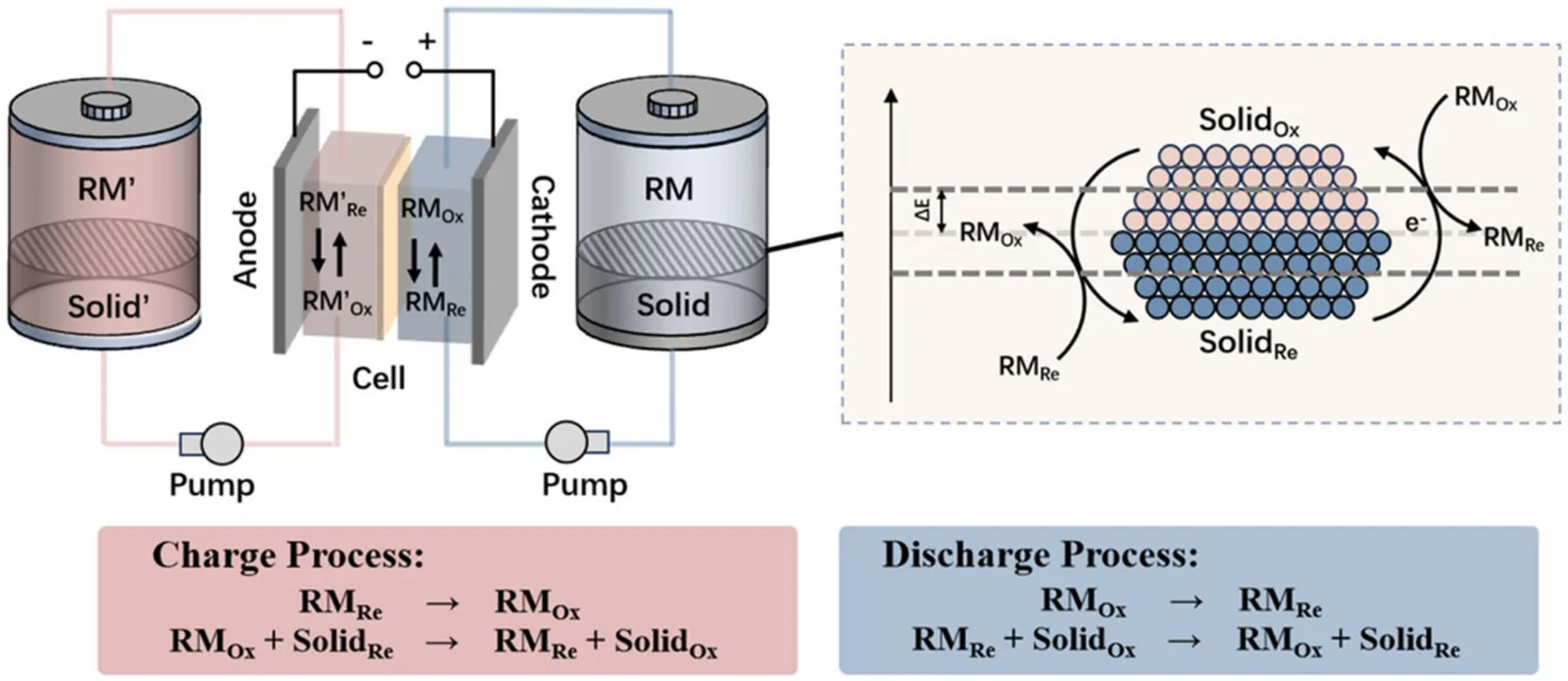
Mechanisms of redox-targeting reactions [94]. Reproduced with permission from the publisher [94].

For most aqueous organic electrolytes, volumetric capacity remains in the 10–100 Ah L^−1^ range, with solubility imposing a fundamental concentration constraint [87]. Redox-targeting reactions offer a way around this bottleneck. Insoluble solid materials fill the storage reservoirs [88,89,90], and the circulating electrolyte needs to carry only a dilute solution of redox mediators [91,92,93]. Each mediator molecule undergoes electrochemical conversion at the electrode before diffusing into the tank and transferring charge to the solid through a chemical reaction (Figure 5). This two-step electrochemical-chemical coupling is what liberates storage capacity from the solubility constraint [94]. What drives these interfacial reactions is not an applied bias but the Nernstian potential gap between the mediator and the solid material [95]. During charging, a reduced mediator whose potential sits below that of the solid donates electrons spontaneously [96]. The reverse holds during discharge, when an oxidized mediator more positive than the solid accepts electrons from it. The first generation of redox-targeting flow batteries relied on dual-molecule designs in which two mediator potentials straddle the solid, one handling reduction and the other oxidation [97,98,99]. Single-molecule redox-targeting instead consolidates both directions into a single molecule with two distinct redox couples, which cuts the molecular inventory in half and reduces parasitic cross-reactivity [39]. Anthraquinone derivatives are unusually versatile in this context because they function effectively on either side of the solid–liquid interface. In one demonstration, Huang et al. select 1,5-dihydroxyanthraquinone as the soluble shuttle and pair it with a poly(anthraquinonyl sulfide)/carbon black composite that supplies the bulk of the storage capacity. The resulting half-cell sustains 573 mAh at 20 mA cm^−2^ for over 1100 h of cycling [38]. A complementary study by Zhang et al. takes a different molecular route, pairing anthraflavic acid and lawsone shuttles with a tribenzophenazine-dione solid. Potential matching to within 18 mV unlocks 80.2% of the theoretical solid capacity, a figure that underscores how critical tight potential alignment is for redox-targeting efficiency [39].

### 5.2. Performance of Anthraquinone-Based Redox-Targeting Flow Batteries

Aqueous organic redox flow batteries frequently grapple with an uncompromising compromise between molecular stability and volumetric energy density. Although synthetic functionalization using sulfonate, carboxylate, or ether chains improves solubility, crowding dissolved quinones above two molar electrons inevitably promotes severe viscosity penalties, sluggish fluid mass transport, and self-dimerization pathways. Redox-targeting flow batteries bypass this solubility limit by redefining the role of dissolved electroactive materials. In these hybrid architectures, soluble anthraquinones do not serve as the exclusive reservoir for electrical charge. Instead, dissolved quinones act as fluidic redox mediators that shuttle electrons between carbon felt electrodes and stationary, high-density solid materials packed within external reservoirs. This operational division of labor decouples stack power output from the volumetric storage capacity of the reservoir tanks. Early targeting designs rely on dual-mediator cocktails to cycle a single solid, using one low-potential molecule for charging and a second high-potential molecule for discharging. Dual-mediator systems introduce chronic thermodynamic overpotentials, reciprocal crossover contamination, and balance-of-plant complexity. Contemporary research consequently prioritizes single-molecule redox-targeting configurations that utilize dynamic Nernstian potential shifts or intrinsic multi-step electron transfer cascades to drive reversible charge exchange through a single molecular shuttle. Table 1 shows the performance comparison of anthraquinone-based flow batteries.

**Figure 6:**
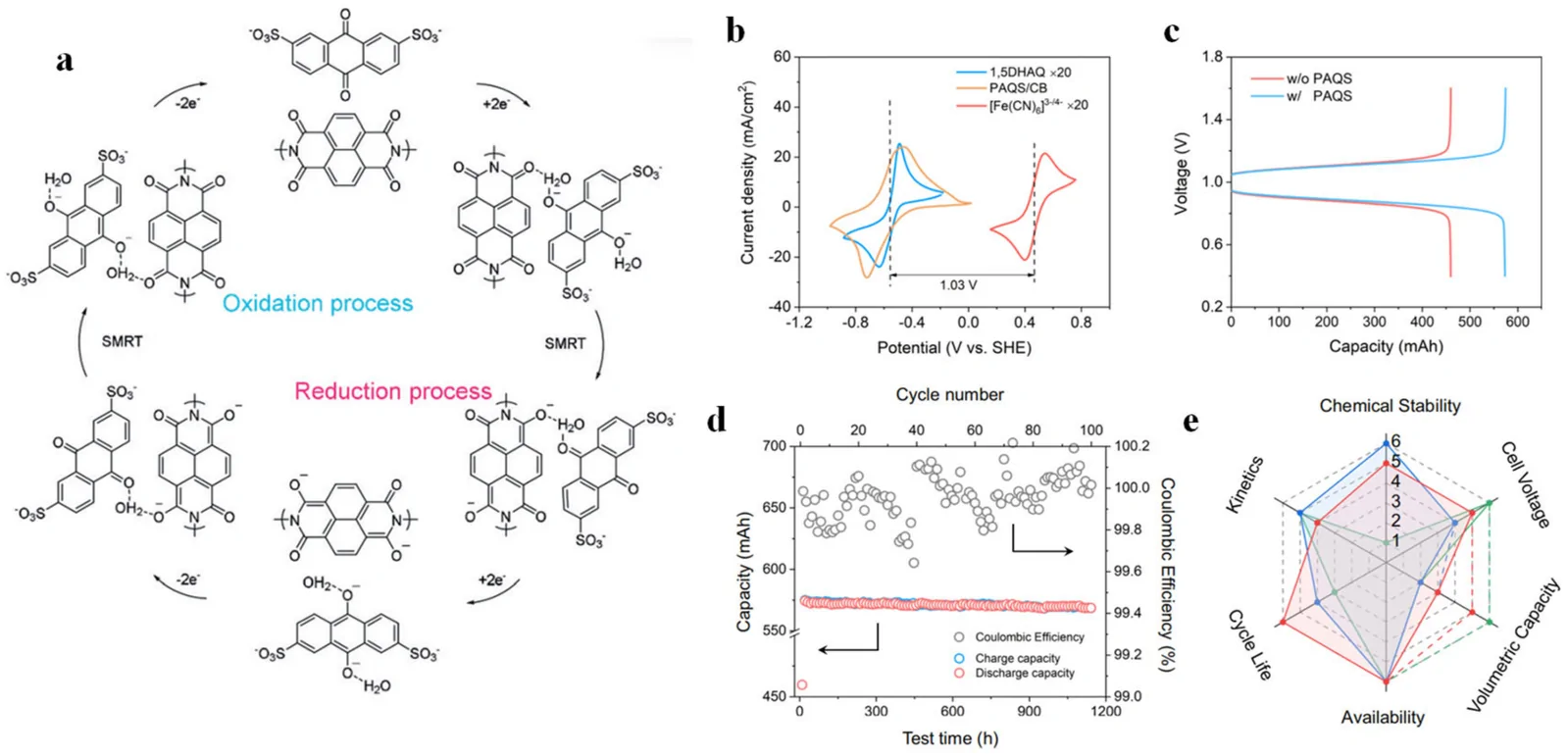
Electrochemical configurations, reaction mechanisms, and cell performance of anthraquinone−based redox−targeting systems. (**a**) Proposed mechanism of hydrogen−bond−mediated charge transfer between 2,7−AQDS and polyimide under pH−neutral conditions [36]. (**b**) Cyclic voltammetry curves of 1,5−DHAQ, potassium ferrocyanide, and the PAQS/carbon black composite recorded in 1 M KOH at a scan rate of 50 mV s^−1^ [38]. (**c**) Cell voltage profiles of the 1,5−DHAQ| [Fe(CN)_6_]^3−/4−^ flow battery in the absence (first cycle) and presence (second cycle) of PAQS/carbon black granules at 20 mA cm^−2^ [38]. (**d**) Extended cycling stability and Coulombic efficiency of the 1,5−DHAQ−PAQS/carbon black targeting system over 1000 h of continuous operation at 20 mA cm^−2^ [38]. (**e**) Comparative evaluation of accessible volumetric capacity, chemical durability, and operational cell voltage among DPivOHAQ, DHBQ, and 1,5−DHAQ−PAQS/carbon black anolyte architectures [38]. Reproduced with permission from the publisher [36,38].

Polymeric organic solids emerge as exceptional solid partners for anthraquinone mediators because their open macromolecular networks facilitate rapid solvated counterion redistribution without the destructive lattice strain that plagues inorganic insertion materials. Zhou and coworkers establish a neutral single-molecule targeting system pairing anthraquinone-2,7-disulfonic acid disodium salt with an insoluble polyimide derived from 1,4,5,8-naphthalenetetracarboxylic dianhydride [36]. Polyimide exhibits a high theoretical specific capacity of 150 mAh g^−1^ and an effective solid electron concentration of 16 M. In this system, 2,7-AQDS operates as the negative shuttle, whereas dissolved sodium iodide pairs with insoluble nickel hexacyanoferrate in the positive circuit. In neutral sodium sulfate, the standard potential of 2,7-AQDS at −0.21 V vs. SHE aligns with the merged electrochemical reduction waves of polyimide located between −0.27 and −0.21 V vs. SHE. The charge transfer pathway between 2,7-AQDS and polyimide relies on a hydrogen-bond-mediated charge transfer mechanism illustrated in Figure 6a. Adsorption of the reduced anthraquinone dianion onto the polyimide backbone through bridging water molecules proves energetically favorable, reducing the reorganization energy of outer-sphere electron transfer and accelerating charge exchange across the solid–liquid boundary. When an acetate buffer moderates localized proton accumulation and raises 2,7-AQDS solubility to 0.2 M, polyimide utilization reaches 83% at 10 mA cm^−2^. The resulting negative reservoir achieves a volumetric capacity of 97 Ah L^−1^, expanding the accessible capacity of unassisted 2,7-AQDS by more than eight times.

Rather than relying entirely on concentration-induced Nernstian potential drifting to drive the solid–liquid reaction, Ma and coworkers uncover a dual-platform single-molecule redox-targeting mechanism that harnesses the intrinsic proton-coupled electron transfer properties of quinone molecules [37]. In this neutral flow system, 2,7-AQDS functions as the mobile electron carrier for solid poly(2,5-dihydroxy-1,4-benzoquinone-3,6-methylene) loaded into the negative storage tank, with ammonium iodide serving as the positive electrolyte. In 1 M Tris buffer at pH 6.8, 2,7-AQDS undergoes a two-electron, two-proton reduction at −0.358 V vs. Ag/AgCl, while the solid PDBM polymer exhibits two distinct, reversible redox waves at −0.23 V and −0.42 V vs. Ag/AgCl that reflect the progressive reduction of neutral quinone to radical monoanion and subsequently to fully reduced dianion. The standard potential of 2,7-AQDS sits directly between these two solid-state redox waves. Galvanostatic charging at 10 mA cm^−2^ produces two distinct voltage plateaus at 0.40 V and 0.70 V. The lower plateau represents the electrochemical reduction of dissolved 2,7-AQDS, whose reduced form chemically reduces pristine PDBM to its radical monoanion intermediate within the tank. Once the reduced shuttle accumulates, the second plateau emerges, driving the further chemical reduction of the radical monoanion to the fully reduced PDBM dianion. Loading PDBM solid into the negative tank expands the discharge capacity from 28.3 mAh for the unassisted solution to 86.4 mAh, demonstrating an exceptional solid material utilization of 91.6% at 10 mA cm^−2^ and 92.5% at 5 mA cm^−2^. The resulting targeting battery maintains 99.9% capacity retention per cycle across 350 h and achieves a volumetric capacity of 97.1 Ah L^−1^, representing a 14.22-fold enhancement over the pristine 2,7-AQDS electrolyte.

In alkaline aqueous environments, dihydroxyanthraquinones frequently suffer from irreversible degradation initiated by the chemical disproportionation of reduced species into anthrone intermediates, followed by oxidative dimerization. Huang and coworkers resolve this fundamental bottleneck by pairing 1,5-dihydroxyanthraquinone with poly(anthraquinonyl sulfide) granules in 1 M KOH [38]. While the common isomer 2,6-DHAQ forms intermolecular hydrogen bonds with surrounding water clusters that accelerate disproportionation, 1,5-DHAQ establishes a localized intramolecular hydration shell that passivates neighboring oxygen atoms, providing chemical stability that is orders of magnitude higher. The cyclic voltammograms in Figure 6b confirm the thermodynamic overlap among dissolved 1,5-DHAQ, the conductive PAQS/carbon black composite, and the potassium ferrocyanide positive couple. Galvanostatic voltage profiles recorded at 20 mA cm^−2^ in Figure 6c demonstrate that incorporating PAQS/carbon black granules into the negative reservoir broadens the discharge plateau, increasing the accessible discharge capacity from 459 to 573 mAh without introducing excessive kinetic polarization. Over 1000 h of continuous cycling, as presented in Figure 6d, the targeting cell delivers an exceptional capacity retention of 99% across 100 cycles, corresponding to a daily degradation rate of 0.02%. The solid PAQS granules act as a protective state-of-charge buffer, continuously scavenging the fully reduced anthraquinone dianions and maintaining the solution state of charge below the critical threshold where dimerization occurs. As shown in the comparative evaluation of Figure 6e, the 1,5-DHAQ-PAQS system achieves a demonstrated volumetric capacity of 47.3 Ah L^−1^ and a theoretical value of 139.5 Ah L^−1^, outperforming conventional formulations like DPivOHAQ and DHBQ in both operational durability and volumetric energy density.

To transcend empirical trial-and-error in finding compatible mediator-solid pairs, Rong and coworkers construct a density functional theory framework based on Gibbs free energy calculations, electrostatic potential distributions, and frontier molecular orbital alignments [40]. When screening substituted anthraquinones against poly(N-anthraquinoyl pyrrole), the authors determine that the number and positioning of hydroxyl substituents modulate the free energy of reaction, identifying 1,8-dihydroxyanthraquinone-2,7-disulfonic acid as an exact potential match for PAQPy at −1.08 V and −1.09 V vs. SHE. Introducing explicit ammonium counterions to mimic realistic hydrogen-bonding environments shifts these potentials positively to −0.50 V and −0.48 V vs. SHE. This positive shift originates from the expansion of the HOMO-LUMO gap and the elongation of electron tunneling distances induced by N-H...O hydrogen bonds. Practical implementation of this computationally predicted couple inside a flow battery produces a 3.8-fold capacity multiplication upon loading PAQPy into the 1,8-DHAQDS reservoir, demonstrating that quantum-mechanical modeling of non-covalent interactions provides a quantitative guide for targeting system design.

Beyond extended polymer networks, small-molecule polycyclic heteroaromatic compounds offer high theoretical charge storage capacities when configured as insoluble tank materials. Zhang and coworkers report an aqueous targeting flow battery utilizing tribenzophenazine-10,15-dione (TBPDO) as a high-capacity solid booster in alkaline media [39]. TBPDO incorporates fused quinone and pyrazine active centers within a rigid planar framework, undergoing two consecutive two-electron, one-proton transfers in 1 M KOH to deliver a theoretical specific capacity of 298 mAh g^−1^ and a solid electron concentration of 23.7 M. To drive the two reduction transitions of TBPDO at −0.714 V and −0.536 V vs. SHE, the authors introduce a dual-shuttle anolyte consisting of 2,6-dihydroxyanthraquinone (anthrafravic acid) and 2-hydroxy-1,4-naphthoquinone (lawsone). In 1 M KOH, the formal potentials of anthrafravic acid (−0.716 V vs. SHE) and lawsone (−0.530 V vs. SHE) match the two respective reduction steps of TBPDO within 6 mV. In a flow cell paired with a ferrocyanide positive electrolyte, loading TBPDO into the negative reservoir increases the discharge capacity from 20.0 to 41.6 mAh at 2 mA cm^−2^, achieving 80.2% solid material utilization and elevating the negative volumetric capacity from 4.0 to 8.3 Ah L^−1^. The cell operates with 80.7% energy efficiency and preserves 99.82% capacity per cycle over 21 cycles at 5 mA cm^−2^, proving that rigid heteroaromatic architectures can function as durable solid boosters in alkaline targeting systems.

### 5.3. Characterizations for Anthraquinone-Based Redox-Targeting Systems

**Figure 7:**
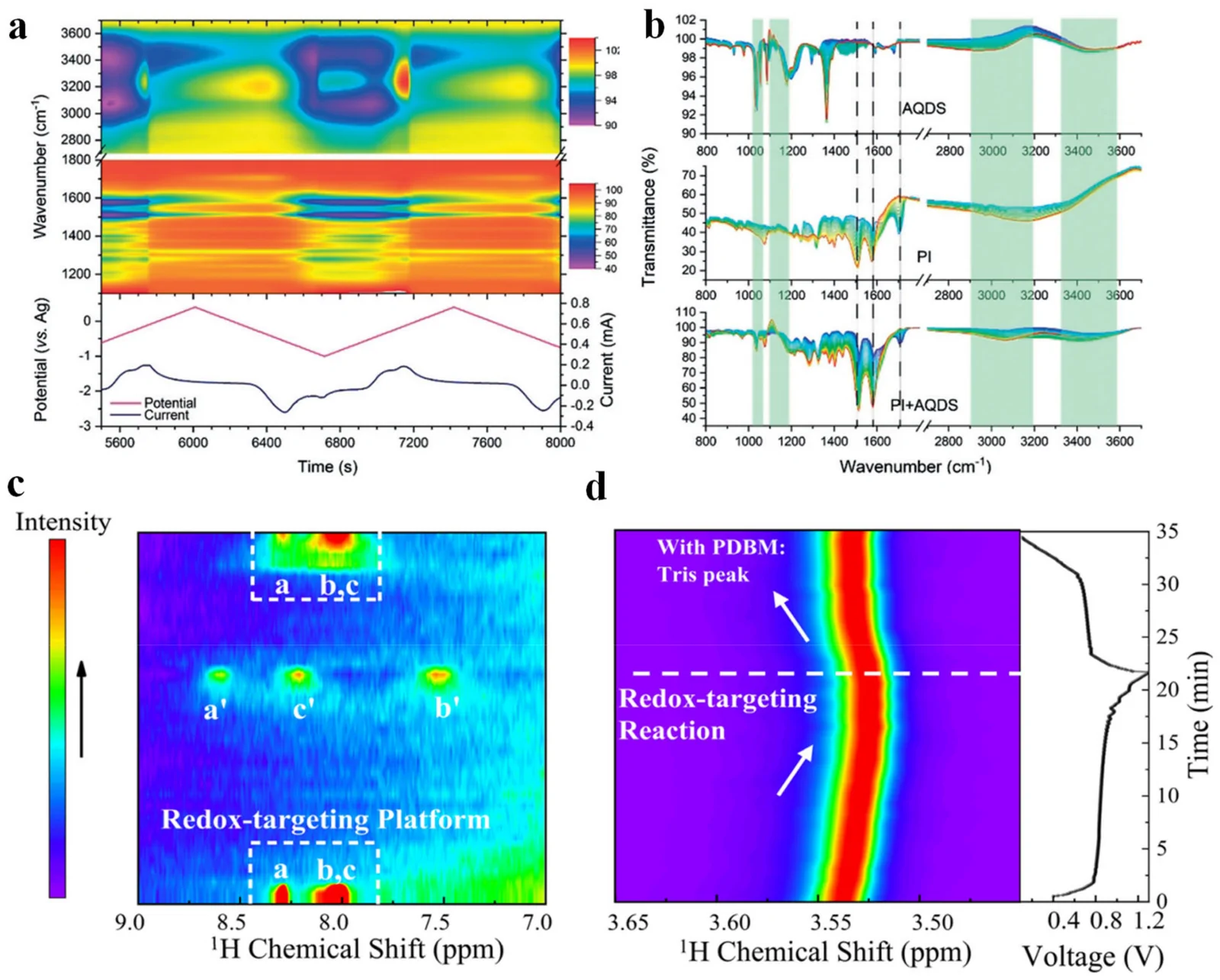
Operando and ex situ spectroscopic characterization platforms for anthraquinone−based redox−targeting flow batteries. (**a**) Dynamic in situ ATR−FTIR spectra of a polyimide thin film undergoing single−molecule redox−targeting reactions with 2,7−AQDS recorded during cyclic voltammetric sweeps in 0.5 M Na_2_SO_4_ [36]. (**b**) Comparative infrared spectral evolution recorded during the reduction of 2,7−AQDS in free solution versus the coupled reduction of polyimide in the presence of 2,7−AQDS [36]. (**c**) Real−time operando ^1^H NMR spectra tracking the resonance signals of dissolved 2,7−AQDS and Tris buffer in a blank flow battery across a complete galvanostatic cycle, The proton resonances for AQDS are labeled as a, b, and c, respectively. [103]. (**d**) Operando ^1^H NMR spectra of 2,7−AQDS and Tris buffer recorded in the AQDS−PDBM/NH_4_I targeting flow battery, demonstrating signal prolongation driven by solid−state charge exchange [103]. Reproduced with permission from the publisher [36,103].

Deciphering the dynamic reactions of redox-targeting systems presents experimental challenges that standard ex situ diagnostic techniques cannot address. Because energy storage occurs inside a packed granular bed separated from the flow stack, researchers must track a multi-stage reaction cascade. This sequence involves heterogeneous electron transfer at carbon felt electrodes, convective transport of dissolved shuttles through fluid channels, interfacial adsorption and electron hopping across solid–liquid boundaries, and phase transformations within the solid grains. Tracking these transient processes without disrupting delicate chemical equilibria requires specialized in situ, operando, and hyphenated spectroscopic tools. Attenuated total reflection Fourier-transform infrared spectroscopy integrated into an operando flow battery provides continuous structural information regarding chemical bonding and solvation structures at the solid–liquid interface. Zhou and coworkers construct a specialized spectra-electrochemical battery to monitor interfacial interactions between dissolved 2,7-AQDS and a polyimide thin film. In this arrangement, a platinum disk working electrode sits within fifty micrometers of an internal reflection crystal, allowing the evanescent infrared beam to detect vibrational modes during cyclic voltammetry sweeps. As shown in Figure 7a, scanning past the reduction potential of 2,7-AQDS causes the imide carbonyl band of polyimide at 1703 cm^−1^ to intensify while adjacent vibrations between 1514 and 1579 cm^−1^ diminish. This spectral modulation reverses during the subsequent anodic scan, demonstrating that solid polyimide undergoes reversible redox transitions driven by dissolved quinone shuttles. The comparative spectra in Figure 7b highlight key differences between 2,7-AQDS reduction in a homogeneous solution and at the polyimide-coated interface. In the presence of polyimide, the carbonyl vibrations of 2,7-AQDS at 1679 and 1364 cm^−1^ blend into the broad polymer absorption profile, while a broad O-H band emerges between 2800 and 3800 cm^−1^. This spectroscopic behavior confirms that a dynamic hydrogen-bonding network bridges the reduced quinone dianion and the polyimide imide groups, facilitating rapid electron transfer across the phase boundary.

The distinction between operando/in situ and ex situ measurements is essential for establishing reliable structure–property relationships in redox-targeting flow batteries. Operando and in situ techniques, including ATR-FTIR, operando ^1^H NMR, UV–Vis spectroscopy, and electron paramagnetic resonance (EPR), enable the evolution of dissolved redox species, transient intermediates, and solid–liquid interfacial interactions to be monitored under controlled electrochemical conditions [68,101]. By avoiding the extraction and transfer of electrolyte samples, these methods minimize sampling-induced alterations in oxidation state, protonation, aggregation, and other solution equilibria, which are particularly relevant to reactive reduced quinone species and radical intermediates. Nevertheless, the measurement configuration itself must be designed to minimize perturbations to the cell environment. In contrast, ex situ characterization, such as solid-state ^1^H NMR analysis of recovered solid active materials, offers greater experimental accessibility and can provide high-resolution information on bulk chemical environments and structural integrity at predefined states of charge. However, the recovered material may undergo chemical relaxation, residual-electrolyte redistribution, or air-induced oxidation during cell disassembly and sample preparation. Consequently, ex situ measurements cannot always preserve the transient chemical states present during operation and should be interpreted alongside operando/in situ results rather than as direct substitutes for them [68,101].

The complementary capabilities of these techniques further enable a more comprehensive understanding of redox-targeting mechanisms. ATR-FTIR provides information on interfacial functional groups, carbonyl vibrations, and hydrogen-bonding interactions, helping elucidate the interactions between dissolved redox mediators and solid active materials. Operando ^1^H NMR can identify and, with appropriate calibration, quantify dissolved mediator species and track their speciation changes during charge and discharge. UV–Vis spectroscopy offers high temporal resolution for monitoring changes in mediator concentration, oxidation state, and reaction kinetics, provided that overlapping absorption features and concentration-dependent effects are appropriately considered. EPR complements NMR by detecting paramagnetic radical intermediates that may be difficult to characterize using conventional NMR measurements. Meanwhile, electrochemical impedance spectroscopy (EIS), combined with distribution-of-relaxation-times (DRT) analysis, helps distinguish ohmic resistance from relaxation processes associated with interfacial charge transfer and mass transport, thereby clarifying the origins of cell polarization and kinetic limitations [68,101,102]. Because DRT inversion and the assignment of individual relaxation processes can be model-dependent, these assignments should be corroborated by appropriate controls and independent measurements.

Operando attenuated total reflectance Fourier-Transform infrared (ATR-FTIR) spectroscopy proves equally valuable for resolving chemical degradation pathways in alkaline media. Huang and coworkers use time-resolved infrared measurements to analyze the distinct stabilities of dihydroxyanthraquinone isomers [38]. For 2,6-DHAQ, holding the electrolyte at open circuit after electrochemical reduction produces new vibrational peaks at 1105 and 1242 cm^−1^, which correspond to the formation of 2,6-dihydroxyanthrone via chemical disproportionation. The delayed appearance of these peaks confirms that water-mediated disproportionation promotes irreversible dimerization. For 1,5-DHAQ, the primary water bending and stretching modes remain stable throughout electrochemical cycling, verifying that its localized hydration shell inhibits self-decomposition. Furthermore, when PAQS/carbon black granules are coated onto the reflection crystal, the reversible appearance and disappearance of a reduced C-O stretching band at 1371 cm^−1^ directly tracks solid-state charge exchange with dissolved 1,5-DHAQ molecules. Nuclear magnetic resonance spectroscopy provides non-destructive, quantitative insights into dissolved mediator speciation and solid-state structural integrity. Conventional offline NMR protocols frequently introduce air contamination during sample handling, distorting the distribution of oxygen-sensitive radical intermediates. Ma and coworkers address this limitation by coupling an operando ^1^H NMR flow apparatus directly to a 400 MHz spectrometer [37]. This platform monitors dissolved 2,7-AQDS and Tris buffer signals throughout continuous cycling. Figure 7c presents operando spectra for a blank flow cell, where aromatic protons a, b, and c shift reversibly from their initial positions at 8.22, 7.95, and 8.02 ppm to 8.58, 7.51, and 8.20 ppm in the fully charged state. At intermediate states of charge, the proton peaks broaden and disappear due to the formation of paramagnetic semiquinone radical anions, reappearing only upon reaching the diamagnetic fully charged or fully discharged states. When solid PDBM is loaded into the reservoir, as shown in Figure 7d, the aromatic proton signals display the same reversible trajectory but undergo substantial temporal broadening. This signal prolongation mirrors the extended voltage plateaus observed during cell cycling, proving that the dissolved quinone maintains a stable intermediate concentration while chemically reducing the solid polymer bed.

Ex situ solution and solid-state ^1^H NMR measurements provide complementary structural verification of insoluble materials recovered at specific states of charge. Zhang and coworkers apply ex situ ^1^H NMR to track the multi-electron hydrogenation of insoluble TBPDO granules across five points along the second galvanostatic cycle [39]. In the corresponding spectra, charging the cell to state C at 1.60 V generates four proton peaks at 8.25, 8.06, 7.77, and 7.52 ppm, which correspond to the fully reduced tetrahydrogenated species TBPDO-4H. Three intermediate peaks at 7.71, 7.56, and 7.03 ppm signal the formation of the dihydrogenated intermediate TBPDO-2H. Discharging the battery to state E at 0.40 V causes the TBPDO-4H peaks to disappear, while the intermediate signals strengthen and then diminish. Post-cycling ^1^H NMR spectra of recovered TBPDO after 21 cycles reveal no extraneous decomposition products beyond trace residual intermediate peaks, confirming that the conjugated phenazine-dione framework remains structurally durable under prolonged cycling. Ultraviolet-visible absorption spectroscopy using flow-through optical cells provides high temporal resolution for quantifying dissolved mediator concentrations. Because oxidized quinones and reduced hydroquinones display distinct electronic transitions, optical absorbance directly measures instantaneous redox conversion. Ma and coworkers implement operando UV-Vis measurements to monitor reduced 2,7-AQDS concentrations at 400 and 520 nm [37]. In a blank battery, the concentration of reduced quinone increases linearly with applied charge. Incorporating solid PDBM transforms this response into two distinct concentration plateaus, confirming that electrochemical reduction at the electrode balances the rate of chemical charge transfer across the solid bed. Correlating these absorbance plateaus with Nernstian potential offsets reveals that the targeting rate slows as the mediator potential approaches the solid potential but accelerates once the second reduction wave provides renewed thermodynamic driving force. Electron paramagnetic resonance spectroscopy complements optical and NMR measurements by detecting open-shell radical intermediates. In both liquid electrolytes and solid polymer granules, EPR spectra quantify radical generation and spin lifetimes. In the PDBM targeting system, ex situ EPR measurements show that radical signal intensity peaks near 50% state of charge and recedes toward fully charged and discharged boundaries, demonstrating that the solid polymer cycles through a stable semiquinone radical monoanion intermediate. Density functional theory simulations link these experimental observations to fundamental thermodynamics. Quantum chemical calculations quantify frontier orbital energies, global electrophilicity indices, and activation barriers along competing reaction pathways. In the computational screening protocol developed by Rong and coworkers [40], electrostatic potential mapping identifies hydrogen-bonding sites between ammonium counterions and quinone oxygen atoms, accurately predicting the potential shifts observed during electrolyte formulation. Differential charge density and Bader charge calculations performed by Ma and coworkers visualize spontaneous electron transfer from hydroquinone hydroxyl groups to polymer carbonyl centers. Combining operando vibrational spectroscopy, real-time magnetic resonance metrology, optical flow analysis, and first-principles modeling establishes a rigorous diagnostic toolkit for designing durable, high-density redox-targeting flow batteries.

## 6. Conclusions and Outlook

This review has systematically summarized the development of anthraquinone-based redox flow batteries (AQ-RFBs), from molecular engineering to advanced redox-targeting (RT) architectures. The unique two-electron redox chemistry, low formal potential, structural tunability, and earth-abundant nature of anthraquinones make them attractive negative electrolytes for aqueous organic flow batteries. Molecular engineering strategies, including substituent introduction, conjugation extension, counterion regulation, and hydrophilic functionalization, have enabled effective tuning of redox potentials and aqueous solubility. Mechanistic studies have clarified the degradation pathways of anthraquinones under concentrated cycling conditions, such as tautomerization, dimerization, side-chain cleavage, and oxidative decomposition. These insights have motivated state-of-charge control, electrochemical rejuvenation, chemical oxidation, and mixed-electrolyte strategies to extend electrolyte lifetime. However, homogeneous AQ-RFBs still face an intrinsic solubility limit, where further increases in concentration often lead to viscosity growth, transport limitations, and accelerated degradation. Redox-targeting flow batteries overcome this limitation by decoupling energy storage capacity from liquid-phase solubility, using dissolved anthraquinone mediators to shuttle electrons between the electrode and high-capacity solid materials. Recent RT systems have demonstrated substantially enhanced volumetric capacity, solid utilization, and cycling stability. Advanced operando and ex situ characterizations, including ATR-FTIR, NMR, EPR, UV-Vis, DRT-EIS, and DFT calculations, have further revealed interfacial charge transfer and reaction mechanisms. Despite these advancements, several critical challenges remain before AQ-RFBs can achieve practical high energy density and long-duration operation.

(1) Green, simple, and fast molecular engineering strategies are needed. Current anthraquinone functionalization often relies on multi-step synthesis, harsh sulfonation or hydroxylation reagents, complex purification, and ion-exchange processes. These routes increase cost, environmental burden, and batch-to-batch variability, limiting scalable deployment. Future work is suggested to develop more sustainable synthetic approaches, such as one-pot reactions, electrochemical or enzymatic synthesis, continuous-flow manufacturing, and atom-economical pathways using low-cost feedstocks. High-throughput computational screening and machine learning can be utilized to accelerate the discovery of high-performance anthraquinone derivatives with favorable redox potential, solubility, and stability.

(2) The cycling stability of anthraquinone-based flow batteries requires more investigation. Degradation is not solely determined by the molecular structure; it is jointly governed by the electrolyte environment, supporting salt, pH, solvent, additive, membrane, electrode, state of charge, cutoff voltage, temperature, and flow conditions. Establishing quantitative relationships among these variables and capacity fade is important in the future. Operando spectroscopy, electrochemical diagnostics, and electrolyte analysis can be integrated to track active species, intermediates, and degradation products. Such mechanism-guided understanding can support adaptive operating protocols, state-of-health monitoring, and controlled electrolyte rejuvenation, enabling degradation to be detected and corrected before irreversible capacity loss occurs.

(3) The construction of redox-targeting flow battery systems remains challenging, and standardized screening principles and evaluation metrics are urgently needed. RTFB performance depends on the thermodynamic potential matching between mediator and solid, interfacial charge-transfer kinetics, solid electronic conductivity, ion accessibility, particle morphology, solid loading, and reactor-level mass transport. Current mediator-solid pairs are often selected empirically. Rational screening criteria and a unified evaluation framework are needed, including redox potential alignment, chemical compatibility, solid utilization, volumetric capacity, energy efficiency, rate capability, and long-term stability. Databases, descriptors, high-throughput DFT/ML workflows, and standardized testing protocols are beneficial to compare different RT systems and guide the design of practical high-energy-density configurations.

(4) The reaction mechanism and solid–liquid interfacial kinetics of RT systems need further clarification. In RTFBs, charge storage involves a multi-step cascade: electrochemical conversion of the mediator at the electrode, convective transport to the reservoir, adsorption at the solid–liquid interface, electron transfer to or from the solid, coupled ion transport, and possible phase transformation within the solid. The rate-determining steps and the roles of hydrogen bonding, counterions, and surface reconstruction remain insufficiently understood. Advanced operando techniques combined with multiscale modeling are encouraged to be employed to resolve interfacial electron-transfer pathways, activation barriers, and transport limitations under real conditions. A quantitative link between molecular-level interactions, interfacial kinetics, solid active-site utilization, and reactor-scale performance is essential for designing durable and efficient RTFBs.

(5) VRFBs remain the most mature technology, but their reliance on vanadium makes active-material cost and supply-chain vulnerability persistent concerns. AQ-based systems can alleviate these issues through earth-abundant organic feedstocks, yet they still lag in practical energy density, cycling stability, membrane requirements, and electrolyte corrosivity. Such comparison clarifies when AQ-based and redox-targeting architectures may offer techno-economic advantages and which performance gaps remain. Commercialization is further constrained by long-term cycling stability, membrane crossover, mediator degradation, solid-reservoir engineering and mass transport, standardization of evaluation metrics, and scale-up of synthesis. Future work should therefore integrate molecular design, interfacial regulation, and device-level optimization to translate laboratory performance into deployable high-energy-density AQ-based redox flow batteries.

## Figures and Tables

**Table 1:** Performance comparison of anthraquinone-based flow batteries.

Reference	System	Volumetric Capacity (Ah L^−1^)	Energy Density (Wh L^−1^)	Cycling Retention	Degradation Rate	RT Solid Utilization
[41]	2,7-AQDS/Br_2_/Br^−^	58.3	50	>92% after 10 cycles (100 h)	0.8%/cycle	N/A
[100]	2,3-DHAQ/[Fe(CN)_6_]^3−/4−^	37.5	17.7	96% after 180 cycles	0.022%/cycle; 0.45%/day	N/A
[28]	PEGAQ/[Fe(CN)_6_]^3−/4−^	80.4	25.2	>90% after 220 cycles (432 h)	0.043%/cycle; 0.5%/day	N/A
[25]	2,6-DHAQ/[Fe(CN)_6_]^3−/4−^	27	18.8	90% after 100 cycles	0.1%/cycle	N/A
[36]	2,7-AQDS + PI/NaI–NiHCF	97	39	65% after 500 cycles	0.0861%/cycle	PI: 83% at 10 mA cm^−2^; 61% at 30 mA cm^−2^; NiHCF; 67% at 10 mA cm^−2^
[37]	2,7-AQDS + PDBM/NH_4_I	97.1	85.45	80% after 200 cycles (350 h)	0.1%/cycle; 0.8%/day	PDBM: 92.5% at 5 mA cm^−2^; 91.6% at 10 mA cm^−2^
[38]	1,5-DHAQ + PAQS/CB/[Fe(CN)_6_]^3−/4−^	47.3	42.1	99% after 100 cycles (1000 h)	0.01%/cycle; 0.02%/day	PAQS/CB: 33% at 20 mA cm^−2^
[39]	2,6-DHAQ + TBPDO/[Fe(CN)_6_]^3−/4−^	10.3	71.9	96.22% after 21 cycles (63 h)	0.180%/cycle; 1.44%/day	TBPDO: 80.2% at 2 mA cm^−2^; 49.3% at 5 mA cm^−2^

## Data Availability

No new data were created or analyzed in this study. Data sharing is not applicable to this article.
